# Efficacy of the transvesical approach for robotic-assisted radical prostatectomy *via* a bladder neck and prostate combined longitudinal incision for the treatment of localized prostate cancer

**DOI:** 10.3389/fsurg.2022.1053140

**Published:** 2023-01-06

**Authors:** YunKai Yang, Jingyun Wang, DaHong Zhang, Qi Zhang

**Affiliations:** ^1^ Department of Graduate Student, Second Clinical Medical School, Zhejiang Chinese Medical University, The 2nd Clinical Medical College, Hangzhou, China; ^2^ Urology & Nephrology Center, Department of Urology, Zhejiang Provincial People’s Hospital, Hangzhou, China; ^3^ Graduate Department, Bengbu Medical College, Bengbu, China

**Keywords:** prostate cancer, robotic surgery, longitudinal incision, radical prostatectomy, L-PARP

## Abstract

**Objective:**

This study explores the feasibility and safety of the transvesical approach of robotic-assisted radical prostatectomy *via* a bladder neck and prostate combined longitudinal incision.

**Methods:**

From June 2017 to May 2021, 41 patients aged from 51 to 69 years underwent the transvesical approach of robotic-assisted radical prostatectomy *via* a bladder neck and prostate combined longitudinal incision (L-RALP). The prostate volume was 22.0–57.8 ml (mean: 36.3 ± 11.1 ml), with a preoperative PSA value of 3.7–12.3 ng/ml (mean: 7.3 ± 1.2 ng/mL). All preoperative Gleason scores were less than or equal to 7 points, and the preoperative TNM stage ranged from T2a to T2b. All patients were diagnosed with prostate cancer by preoperative prostate biopsy or postoperative pathological specimens after prostatectomy. The operation, blood loss, hospitalization, erectile function and postoperative urinary continence were recorded. Patients were defined as continent if they answered “zero pad” per day, and they were invited to fill out The International Consultation on Incontinence Questionnaire (ICI-Q-SF) after the catheter removal at 4 and 24 weeks.

**Results:**

All the operations were completed by robotic-assisted radical prostatectomy without transition to open surgery. The surgery time was 105–131 min (mean: 111.3 ± 14.9 min), with an estimated blood loss of 50–220 ml (mean: 95.5 ± 27.3 ml). The postoperative hospital stay was 3–8 days (mean: 5.2 ± 1.7 days), and the postoperative catheter was removed after 5–7 days (mean: 6.3 ± 1.1 days). After 24 weeks of follow-up, 35 cases (85.4%, 35/41) obtained immediate urinary continence after the catheter removal in 24h. All patients had regained continence 24 weeks postoperatively (100%, 41/41).

**Conclusion:**

The transvesical approach of robotic-assisted radical prostatectomy *via* a bladder neck and prostate combined longitudinal incision is a safe and effective surgical technique, beneficial for early continence recovery and erectile function, and it is also suitable for prostate cancer patients after prostate enucleation.

## Introduction

Prostate cancer (PCa) is the most common malignancy among older men worldwide and the second leading cause of cancer-related morbidity and mortality in the Western world, with an increasing incidence and mortality year by year (1–3). Radical prostatectomy (RP) is an effective and preferred treatment method for localized prostate cancer. Robot-assisted laparoscopic surgery (RALP) is now widely used in clinical practice because of the highly flexible robotic arm, extremely precise operating instruments, and clearer surgical field of vision (4, 5). Furthermore, RALP can avoid damage to the surrounding prostate tissues, reduce intraoperative bleeding, better preserve functional nerves, reduce the positive rate of surgical margins, and improve the quality of patient prognosis (6–9).

Some studies have shown that the positive rate and recurrence rate of conventional RALP were significantly lower than laparoscopic radical prostatectomy (LRP), but the recovery of urinary continence and erectile function were unsatisfactory (10). Retzius-sparing RALRP *via* posterior approaches has advantages for localized prostate cancer, such as early urinary continence recovery, erectile function recovery, and higher quality of life (11, 12). However, most urological clinicians anterograde separate the prostate by the anterior approach because the anatomical landmarks are well known to them, and the whole procedure is performed under a relatively good field of vision and space. A new surgical technique is the transvesical approach of robotic-assisted radical prostatectomy through the bladder neck and prostate combined longitudinal incision (L-PALP). This approach does not open the pelvic fascia and preserves the important tissue structures around the prostate, and it has a good surgical field under the longitudinal incision. Moreover, it has unique advantages in preserving erectile function and urinary continence in patients with prostate cancer after surgery. Therefore, this study investigated the efficacy and safety of the intrafascial radical prostatectomy through the bladder, neck, and prostate combined longitudinal incision to provide new surgical options for prostate cancer patients.

## Materials and methods

### Study population

According to the Pasadena Consensus formulated in 2012 recommended “0-pad” as the definition of no urinary incontinence (UI) in patients with prostate cancer after surgery, patients were defined as continent if they answered “zero pad” per day (13).

Inclusion criteria: 1. All patients were diagnosed with prostate cancer by preoperative prostate biopsy or postoperative pathological specimens after prostatectomy. 2. All preoperative Gleason scores were less than or equal to 7 points, and none of the patients received hormonal therapy. 3. The preoperative TMN staging of all patients was within T2c, and imaging examination did not suggest lymph node metastasis or distant-metastasis 4. All patients have no personal history of malignancy. 5. All patients' preoperative initial international index of erectile function 5 scores (IIEF-5) were more than 16 points. 6. Patients with urinary incontinence (UI) will be excluded.

A total of 41 patients aged 51 to 69 with localized prostate cancer were included in this study. The prostate volume was 22.0–57.8 ml, and the median volume was 36.3 ± 11.1 ml. The preoperative PSA value was 3.7–12.3 ng/ml, the median PSA was 7.3 ± 1.2 ng/ml, the preoperative Gleason scores were ≤7 points, and re-TNM staging T2a–T2b. The mean preoperative IIEF-5 score of all patients was 18.9 points.

### Medical team infromation

All procedures were performed by the same clinical urologist and the same assistant, the surgeon had more than 10 years of working experience and had performed more than 300 RALP before this study. And perioperative care provided by the same nursing team as well.

### Surgical procedure

#### Posture, channel establishment, and robotic arm placement

All surgeries were performed through the abdominal cavity and assisted by robots. Under general anesthesia, the legs were separated and fixed in the 30° Trendelenburg position. Five cannulas were placed, with a 12 mm cannula placed in the longitudinal incision below the umbilicus as the observation hole, and the pneumoperitoneum pressure was maintained at 12–14 mmHg (1 mmHg = 0.133 kPa). Under direct vision, an 8 mm cannula was placed 7 cm to the right of the mirror hole to connect to the robot. For the first robotic arm, an 8 mm sleeve was placed 7 cm to the right to connect to the third robotic arm, and an 8 mm sleeve was placed 7 cm to the right of the observation mirror hole to connect to the second robotic arm, and then a 12 mm casing was placed 8 cm to the left as an auxiliary hole.

#### Surgical process

**Figure 1:**
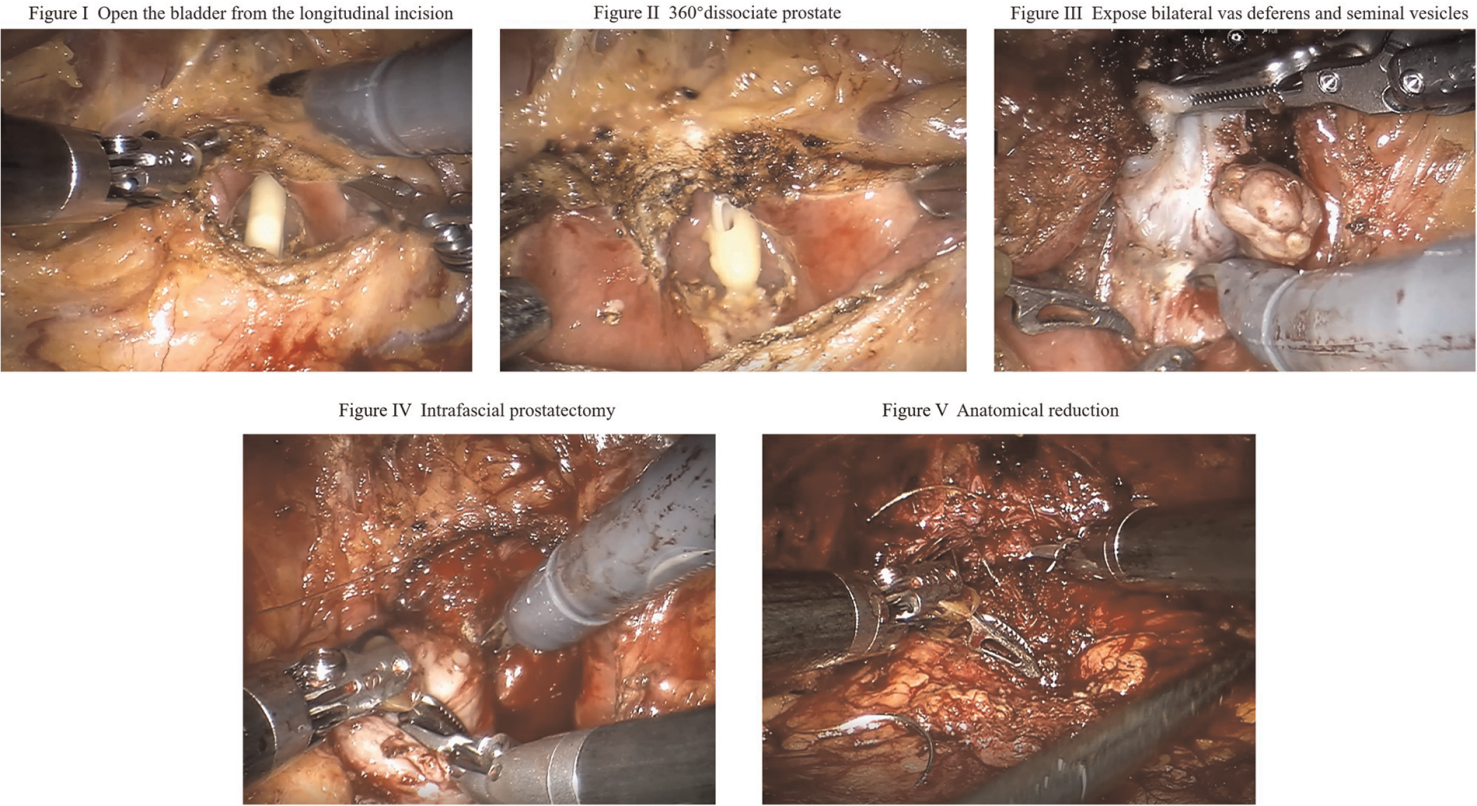
Surgical procedure.

(1) First, space was established around the prostate by opening the peritoneum at the first peritoneal fold on the back of the bladder; Then, the connective tissue in the retroperitoneal space of the bladder was dissociated to the retropubic space before the adipose tissue above the prostate was cleaned with electrocautery to expose the pubic prostate.
(2) The catheter was pulled to determine the position of the bladder neck (Figure 1A), and a longitudinal bladder incision (3–5 cm) was made on the bladder neck and prostate (Figure 1B). The bladder was opened to expose the neck, and a 360° incision was made around the bladder neck. The ureteral opening was identified, and the incision was removed from the ureteric opening. The posterior lip of the bladder neck was opened, and the incision was expanded to the 5th and 7th points of the bladder neck along the outer posterior border of the prostate capsule, and the bladder prostatic muscle was incised to expose the bilateral vas deferens and seminal vesicles (Figure 1C).
(3) The prostate tissue was lifted from the bilateral vas deferens and seminal vesicles were separated. The posterior wall of the prostate in front of Denonvilliers’ fascia was opened to expose the prostate capsule and expand the separation plane between the rectum and prostate to the lateral ligaments of the prostate. An electric energy knife was used to stop bleeding. The inner plane of the prostatic fascia was separated, the lateral prostatic ligament was dissociated to the apex of the prostate, and NVB was retained in the fascia. If necessary, a 4-0 barbed suture was used to stop the bleeding.
(4) The pneumoperitoneal pressure was increased to 18–20 mmHg before the plane was separating at the inner fascia layer (Figure 1D) from both sides of the prostate capsule to the apex. Without cutting the puboprostatic ligament, the urethra was exposed using an electric incision along the prostate capsule to cut off the attachment of the DVC. Scissors were used to cut the anterior wall of the urethra sharply, exit the catheter, and then cut the posterior wall of the urethra, taking care to retain a sufficient length of urethral tissue length. The prostate was removed and checked to ensure that the prostate capsule was intact. If there is blood oozing occurs on the wound surface, the urinary catheter can be pulled to compress the wound surface and stop bleeding.
(5) Double-needle barbed line anastomosis of the bladder and urethra: a 3–0 double-needle single-Joe barbed line anastomosis was started at the 6 o'clock positionreconstruct the bladder neck. The perineum can be lifted to better expose the urethral stump and reduce the tension of vesicourethral anastomosis. After 2–3 stitches were sutured on both sides of the posterior wall, the sutures were tightened to completely close the posterior wall of the vesicourethral anastomosis, and the ends of the two sutures continued to be sutured clockwise and counterclockwise, and the bladder incision was closed. During reconstruction, it is important to avoid damage to or stretching of the ureteral orifice. The anterior bladder tissue was closed, an anatomic reduction was performed (Figure 1E), the F18 double-lumen urinary catheter was replaced, the pelvic drainage tube was placed, and the specimen was taken.

## Results

The operation time ranged from 105 to 131 min, with a median duration of 111.3 ± 14.9 min. Intraoperative blood loss ranged from 50 to 220 ml, with a median of 95.5 ± 27.3 ml. The postoperative hospitalization time was 3–8 days (median: 5.2 ± 1.7 days), and the postoperative catheter indwelling time was 5–7 days (median: 6.3 ± 1.1 days). There were no serious surgical complications.

Postoperative pathology showed that 27 patients had a Gleason score of 6 points, 12 had a Gleason score of 7 points, and 2 had a Gleason score of 8 points. Postoperative TNM staging ranged from T2a–T2c. Two cases of prostate apex had a positive resection margin after the procedure, and the positive rate of resection margin was 4.88% (2/41). The patients were recommended to undergo external radiation therapy. All patients showed good recovery after six weeks of follow-up, and the PSA of 40 patients was <0.2 ng/ml. One patient's PSA was 0.85 ng/ml, and it was maintained below 0.2 ng/ml after external radiation therapy. The patients were followed up for 24 weeks, and each enrolled patient was required to complete a rigorous Kegel training for 24 weeks.; 35 cases (85.4%, 35/41) obtained immediate urinary continence after the catheter removal in 24h, 4 had no urine leakage and achieved continence at 1 week after the catheter removal. 2 had no urine leakage and achieved continence at 4 weeks after the catheter removal. All patients had regained continence 24 weeks postoperatively (100%, 41/41). As for ICI-Q-SF (14), the mean score of all patients were below 4 points both 4-week or 24-week after the catheter removal. After 6 months follow-up, All patients have no severe erectile dysfunction. As far as IIEF-5 score is concerned, there were no statistically significant differences between the preoperative and postoperative data (*P* = 0.141). (Table 1)

**Table 1:** Perioperative outcomes.

Perioperative outcomes (*n* = 41)	mean ± sd (*n*%; IQR)	*P*
operation time (min)	111.3 ± 14.9	
Intraoperative blood loss (ml)	95.5 ± 27.3	
postoperative hospitalization time (day)	5.2 ± 1.7	
surgical complications	–	
Gleason score		
6	27.0 (65.8%)	
7	12.0 (29.2%)	
8	2.0 (5.0%)	
TNM stage		
T2a	24.0 (58.5%)	
T2b	12.0 (29.2%)	
T2c	5.0 (12.2%)	
24h urinary continence	35.0 (85.3%)	
6-month urinary continence	41.0 (100%)	
ICI-Q-SF (4 week)	3.0 (2.0, 5.0)	
ICI-Q-SF (24 week)	2.0 (2.0, 6.0)	
preoperative IIEF score	18.9 ± 2.4	*P* = 0.141
6-month postoperative IIEF score	18.0 ± 2.7	

ICI-Q-SF, International Consultation on Incontinence Questionnaire; TNM, tumor node metastasis classification; IIEF, initial international index of erectile function 5 scores.

## Discussion

The vascular and nerve bundles around the prostate, the length of the urethra and the surrounding sphincter, the puboprostatic ligament, the pudendal artery, and the prostatic venous plexus are the anatomical structures related to the maintenance of sexual function and urinary continence after surgery. Traditional RALP techniques are based on the anatomy of the previous open retropubic approach, which may cause damage to these anatomical structures, causing postoperative sexual and urinary continence abnormalities (15–18). The Italian team of Professor Bocciardi proposed that the RALP technique that preserves the Retzius' space can effectively avoid damage to these anatomical structures during surgery and achieve good surgical results. This new surgical procedure is known as the posterior approach RALP or RALP with preserved Retzius' space (19, 20).

However, there are two main technical difficulties in the posterior approach RALP: (1) A narrow operating space: the larger the prostate volume, the narrower the operating space. (2) Anastomosis of the bladder and urethra: since the periprostatic fascia is not separated in the posterior approach RALP, there is tension in the anastomosis between the urethra and the urethra, and the bottom-up reverse field of view is required during the anastomosis process requiring the clinician's suturing skills (21, 22).

Given these operational difficulties, intrafascial radical resection of the prostate through an anterior approach was performed to preserve important urinary continence-related tissue structures in the Retzius space. We completed 41 cases of L-RALP, including patients after enucleation of the prostate, demonstrating that this surgical method has the following advantages: (1) It does not require the liberation of the bladder and the prevesical space, and the operation is limited to the deep pelvic space around the prostate. The prostate can be completely resected within the fascia, and the integrity of the vascular and nerve bundles can be fully preserved, which could reduce the damage of radical prostatectomy as much as possible; (2) Intraoperative bleeding is reduced by suture ligation, avoiding the influence of thermal injury on long-term sexual function and urinary continence function; (3) The integrity of the puboprostatic ligament and pudendal artery is preserved; (4) A longitudinal incision is used to open the bladder to more easily expose the vas deferens and seminal vesicles, the separation steps of the bladder neck are reduced, and the damage to the detrusor muscle group is minimized; (5) The bladder neck is easy to identify and retain during the operation, which reduces the incidence of bladder neck contracture and ureteral orifice injury, shortening the indwelling time of the postoperative catheter; (6) The anatomy of the L-RALP starts from the 6 o'clock position of the prostate because the Denonvillier fascia is thicker here, and it is easy to separate with scissors, which can completely preserve the outer fascia and NVB on both sides of the prostate; (7) A subumbilical incision and 0° mirror can be used for the whole operation. At an extreme angle, a 30° mirror can be considered, or the observation hole mechanical arm raised to improve the field of vision.

Although this surgcial approach is feasible at laparoscopic radical prostatectomy theoretically, only patients with small volume of prostate and localized prostate cancer are suitable for this approach. Small surgcial space and narrow surgcial visual field limit surgeons' laparoscopic operate. Compared to L-RALP, transvesical approach of laparoscopic radical prostatectomy *via* a bladder neck and prostate combined longitudinal incision may need a surgeon with extensive clinical experience to perform.

## Conclusion

The transvesical approach of robotic-assisted radical prostatectomy *via* a bladder neck and prostate combined longitudinal incision (L-PALP) is technically feasible, protecting the anatomical structures around the prostate related to sexual function and urinary continence during the operation. It is especially suitable for localized prostate cancer that requires high urinary continence and erectile function, as well as prostate cancer patients after enucleation. The continuous popularization of domestic robotic equipment for precise surgcial treatment of prostate cancer is the ultimate goal pursued by all urologists.

## Data Availability

The raw data supporting the conclusions of this article will be made available by the authors, without undue reservation.
